# Thermostability mechanisms of β-agarase by analyzing its structure through molecular dynamics simulation

**DOI:** 10.1186/s13568-022-01394-x

**Published:** 2022-05-06

**Authors:** Lixing Liu, Lixi Cai, Yunmeng Chu, Min Zhang

**Affiliations:** 1 College of Basic Medicine, Putian University, Putian, 351100 Fujian China; 2 Putian University Key Laboratory of Translational Tumor Medicine in Fujian Province, Putian, 351100 Fujian China; 3 Department of Bioengineering and Biotechnology, Huaqiao University, Xiamen, 361021 Fujian China

**Keywords:** Thermostability, β-agarase, Molecular simulation, RMSD, RMSF, Salt bridge

## Abstract

Agarase is a natural catalyst with a good prospect in the industry. However, most of the currently discovered β-agarases are unsuitable for relatively high-temperature and high-pressure conditions required by industrial production. In this study, molecular dynamics simulations were first used to investigate the dynamic changes of folding and unfolding of mesophile and thermophile β-agarases (i.e., 1URX and 3WZ1) to explore the thermostability mechanism at three high temperatures (300 K, 400 K, and 500 K). Results showed that the sequence identity of 3WZ1 and 1URX reaches 48.8%. 1URX has a higher thermal sensitivity and less thermostability than 3WZ1 as more thermostable regions and hydrogen bonds exist in 3WZ1 compared with 1URX. The structures of 1URX and 3WZ1 become unstable with increasing temperatures up to 500 K. The strategies to increase the thermostability of 1URX and 3WZ1 are discussed. This study could provide insights into the design and modification of β-agarases at a high temperature.

## Introduction

Agarase is a hydrolase that catalyzes the hydrolysis of glycosidic bonds in the agarose molecule. Based on the different types of catalyzed glycosidic bonds, they can be divided into α-agarase (E.C. 3.2.1.158) and β-agarase (E.C. 3.2.81) (Chi et al., 2012). The vast majority of currently discovered agarases are β-agarases, mainly from marine microorganisms, such as *Pseudomonas* (Lee et al., 2000), *Pseudoalteromonas* (Lee et al., 2000; Oh et al., 2010), *Catenovulum* (Xie et al., 2013), *Vibrio (*Dong et al., 2007*)*, and *Flammeovirga* (Chen et al., 2019). Agarase can be used to isolate and prepare algal protoplasts and extract unsaturated fatty acids, vitamins, carotenoids, betaine and other bioactive substances from algae (Chi et al., 2012). Given the importance of this enzyme, people have increased the mining of this enzyme resource from the ocean to obtain more agarases.

Although more and more agarases have been discovered, few can be utilized in industrial applications. This is mainly because this kind of enzyme fails to adapt to the relatively high temperature and high pressure, strong acid and alkali conditions required by several industrial production processes, though few agarases with good thermostability were reported (Ramos et al., 2018; Hafizah et al., 2019; Zhu et al., 2019). Currently, targeted enzyme modification with high efficiency can be performed by genetic engineering to potentially increase the environmental adaptation of agarases. However, it is necessary to understand the characteristics of agarases in high temperatures and the corresponding thermostability mechanism from the molecular level to obtain meaningful data for the targeted enzyme modification.

Currently, the stability mechanism of enzymes cannot be explained by an existing theory due to complex mechanisms impacted by various factors such as the composition of amino acids, disulfide bridges, the hydrophobic core, and the salt bridge (Lehmann et al., 2000). Molecular dynamics (MD) simulation can use microscopic computational information to provide insights into thermal enhancing and reducing factors (Karplus and McCammon, 2002). This strategy has been a well-known method to understand the structure and properties of numerous enzymes (Sárosi and Lybrand, 2018; Chen et al., 2021; Ghattas et al., 2020; Vahed et al., 2020; Kumar et al., 2021). However, MD has rarely been employed in β-agarase engineering to provide detailed descriptions of β-agarase folding and unfolding thermal motion at different temperatures.

In this study, we selected two β-agarase monomeric crystal structures belonging to the glycoside hydrolase (GH) family 16, one is the mesophile 1URX and the other is the thermophile 3WZ1, based on the homology with different optimum temperatures. MD simulations of these two crystal structures were performed at 300 K, 400 K, and 500 K (Berman et al., 2000). The conformational dynamics, amino acid fluctuation, and the number of formed salt bridges and hydrogen bonds of the two enzymes at different simulated temperatures were obtained. To our knowledge, we firstly used MD simulation to study the thermostability of β-agarases for understanding the thermostability mechanism. We believe it would help build new design strategies for enhancing the thermostability of β-agarase to highlight possible future applications in the industry.

## Materials and methods

### Materials

The β-agarase crystal structures used in this paper were obtained from the enzyme database of the Protein Data Bank (PDB, https://www.rcsb.org/) (Berman et al., 2000); the PDB codes are 1URX (Allouch et al., 2004) and 3WZ1 (Takagi et al., 2015), isolated from *Zobellia galactanivorans* (Hehemann et al., 2010) and *Microbulbifer thermotolerans* JAMB-A94 (Takagi et al., 2015), respectively. The mesophile microorganism, *Zobellia galactanivorans* (formerly known as *Cytophaga drobachiensis*), has a growth temperature range of 13–45 ℃, with the optimum temperature at 35 ℃ (Barbeyron et al., 2001; Groisillier et al., 2015). As a deep-sea bacterium, the thermophile microorganism, *Microbulbifer thermotolerants* JAMB-A94, has an optimal growth temperature of 43–49 ℃. Moreover, the cloned and expressed β-agarase from this strain has high enzyme activity at 60 ℃, which is currently the most thermostable β-agarase with a 3D structure (Takagi et al., 2015). Therefore, two types of β-agarases, 1URX and 3WZ1, are chosen as representatives of mesophile and thermophile enzymes, respectively. The specific original structures and corresponding residues were then identified by carrying out pairwise sequence alignment using ClustalW (Thompson et al., 1994) and structural alignment using ESPript server (Gouet et al., 1999) and Visual Molecular Dynamics (VMD) v1.6 (Humphrey et al., 1996). The Q_H_ value was calculated for assessing structural homology and structural conservation of 1URX and 3WZ1.

### MD simulation

MD simulations for β-agarases were performed using NAMD v2.9 package with the CHARMM27 force field (MacKerell Jr et al., 2000). The simulation systems were in the canonical (NVT) ensemble. The system was solvated by adding transferable intermolecular potential with three interaction sites (TIP3P) water molecules and neutralized by using VMD. The electrostatic energy was calculated in Particle Mesh Ewald (PME) method (10.1002/1096-987X(200,009)21:12<1049::AID-JCC3>3.0.CO;2-F). The simulation time step was 2.0 fs. For every 500 steps, the specific output at the corresponding frame was obtained. A maximum of 30,000 steps were carried out for achieving energy minimization. The corresponding structure was regarded as original structure for 1URX and 3WZ1 at the zero simulation time.

The two enzymes (1URX and 3WZ1) were placed in aqueous solution for MD simulation to investigate the structural stability of 1URX and 3WZ1 at different temperatures, i.e., 300 K, 400 K, and 500 K. Simulations were conducted for 5 ns (i.e., 5000 ps) at each temperature using the following indexes: the root-mean-square deviation (RMSD) and root-mean-square fluctuation (RMSF), solvent accessible surface area (SASA), Van Der Waals (VDW) force, the average number of protein–protein and protein–solvent intermolecular hydrogen bonds. Note that RMSD is a commonly used quantitative measure of the similarity between the protein conformation and its original structure at a specific time. Root mean square fluctuation (RMSF) refers to the root mean square displacement of each amino acid in a frame conformation compared with the average conformation.

In addition, the evolution of secondary structures of 1URX and 3WZ1 with simulation time were estimated to further understand the impact of temperatures on the structural stability of 1URX and 3WZ1. The RMSF and RMSF-difference values were calculated and analyzed. The RMSF-difference values at 400 K (500 K) are obtained by subtracting the RMSF values at 300 K from the corresponding RMSF values at 400 K (500 K). If the RMSF-difference of a residue is larger than its corresponding average RMSF-difference for all residues, then we define it as a thermal sensitive residue, indicating the corresponding residue is instable. Otherwise, we define it as a thermostable residue, indicating the corresponding residue is stable.

Finally, the number of hydrogen bonds and salt bridges as well as the distance of salt bridges were calculated through MD simulations to explore the intramolecular interactions for 1URX and 3WZ1.

## Results

### Sequence alignment and structural comparison

**Fig. 1:**
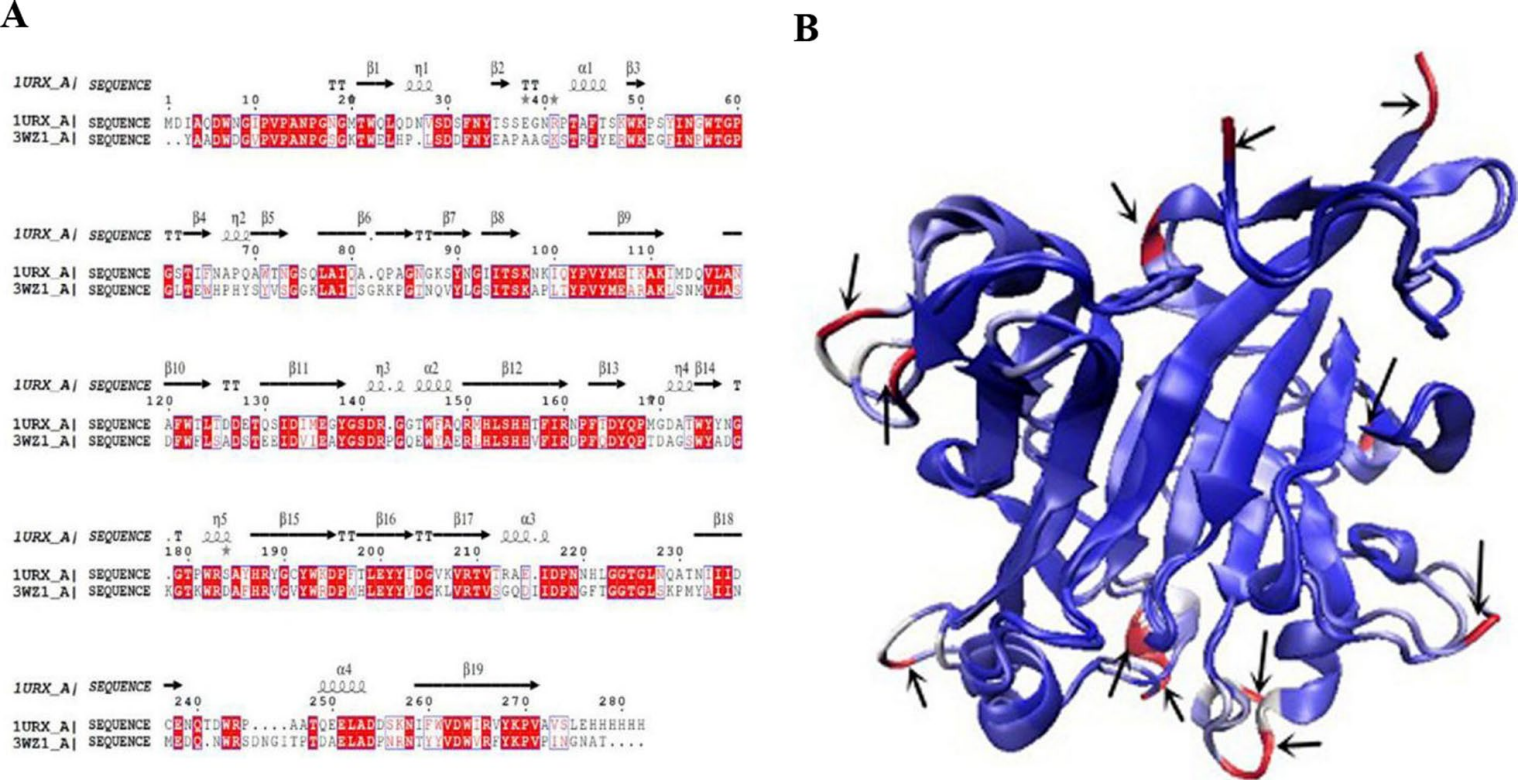
Sequence alignment (**A**) and structure alignment (**B**) of two β-agarases (1URX and 3WZ1)

Sequence alignment of the two β-agarases revealed that 3WZ1 showed 48.8% sequence identity with 1URX. The secondary structure elements are also shown in Fig. 1A, which demonstrates that 1URX has one more α helix (residues 22–24) and one more β sheet (residues 31–33) but one less α helix (residues 240–245) compared to 3WZ1. We also compared the structures of the two proteins by MultiSeq in VMD prior to the heating simulations in 300 K, 400 K, and 500 K, shown in Fig. 1B. The Q_H_ value was 0.8758. This high value is expected as both 1URX and 3WZ1 are from the subfamily 16 in glycoside hydrolase family GH16 (GH16_16) (Naretto et al., 2019) and thus they have similar structural features.

We also colored the molecules according to the Q value of each residue (Q_res_). As shown in Fig. 1B, the blue areas refer to the molecules with Q value less than 15% difference, indicating they are homologous in structure. While the red areas (highlighted by the arrows) refer to the molecules with Q value larger than 15% difference indicating they are not homologous in structure and could differ each other in structures. Statistically, we found that there are four different sites (Asn23, Asn36, Gly140 and Thr237) in 1URX and eight different sites or regions (Ala1, Pro33, Gly82, Pro140 ~ Gly141, Lys177, Gln213, Ser244 ~ Asp247 and Ile276 ~ Asn277) in 3WZ1. The different characteristics of sites may cause impact on the thermostability mechanism of 1URX and 3WZ1 at high temperatures. This speculation is tested through our following MD simulation in next sections.

### Structural stability of the proteins at different temperatures

**Fig. 2:**
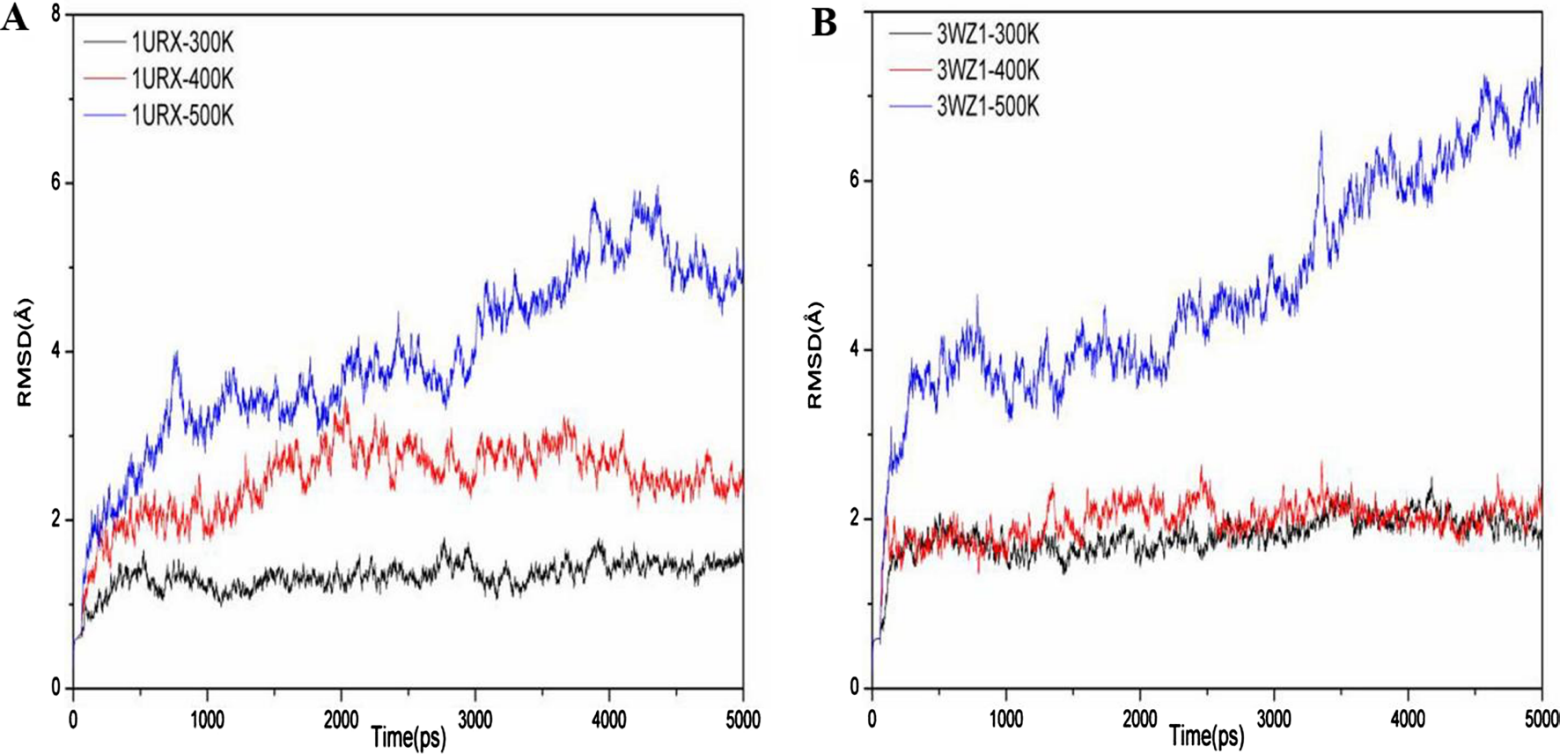
Backbone RMS deviation values of (**A**) 1URX and (**B**) 3WZ1 at different temperatures as a function of time. The duration of each simulation is 5 ns

The RMSD values of the backbone for proteins 1URX and 3WZ1 at three parallel simulation temperatures are respectively shown in Fig. 2A and B. The result showed the RMSD values initially increase and stabilize at 300 K for both 1URX and 3WZ1. The average RMSD values are close to each other for 1URX and 3WZ1 (1.34 Å and 1.80 Å), respectively (Table 1). Compared to 300 K, the RMSD values have a similar fluctuation with the elapsed simulation time for 1URX and 3WZ1 at 400 K. However, the average RMSD value for 1URX at 400 K is 2.47, which is much higher than that at 300 K. Interestingly, this difference is not observed in 3WZ1. For the temperature of 500 K, the RMSD values of both 1URX and 3WZ1 increased significantly with the elapsed simulation time.

**Table 1:** The average values of SASA, VDW energies and the average number of protein-protein (intramolecular) and protein-solvent H-bonds in 1URX and 3WZ1 at different temperatures

	300 K	400 K	500 K
1URX			
RMSD	1.34	2.47	3.94
RMSF	1.27	2.10	3.55
SASA	109.33	130.17	190.38
Protein–protein H-bond	69.94	54.55	45.88
Protein-solvent H-bond	199.77	145.02	97.32
VDW-force (protein)	− 1072.66	− 924.63	− 717.41
VDW-force (protein-water)	− 459.07	− 427.31	− 394.84
3WZ1			
RMSD	1.80	1.97	4.74
RMSF	3.19	1.96	4.22
SASA	120.91	146.69	209.96
Protein–protein H-bond	76.83	61.18	47.85
Protein-solvent H-bond	216.39	155.81	112.68
VDW-force (protein)	− 1027.51	− 875.258	− 662.48
VDW-force (protein-water)	− 474.68	− 418.29	− 426.44

SASA, H-bonds, VDW-force denote solvent accessible surface area, hydrogen bonds and Van Der Waals force, respectively. The values of SASA and VDW-force are given in Å and kcal/mol respectively

**Table 2:** The average distance (Å) of salt bridges in 1URX and 3WZ1

	Salt bridge	300 K	400 K	500 K
1URX	Asp26-Lys44	3.3	3.4	3.5
	Asp200-Arg184	6.2	3.9	5.2
	Asp250-Lys253	5.2	4.1	4.4
	Asp122-Lys95	3.0	2.9	3.0
	Glu34-Arg37	3.5	7.0	4.3
3WZ1	Asp168-Arg208	3.4	3.8	8.4
	Glu60-Lys80	3.2	3.5	6.7
	Glu127-Lys229	4.1	3.0	2.9
	Asp252-Arg79	3.5	3.6	4.8
	Asp257-Arg260	4.0	4.4	7.7
	Glu128-Arg158	9.8	4.5	3.9

**Fig. 3:**
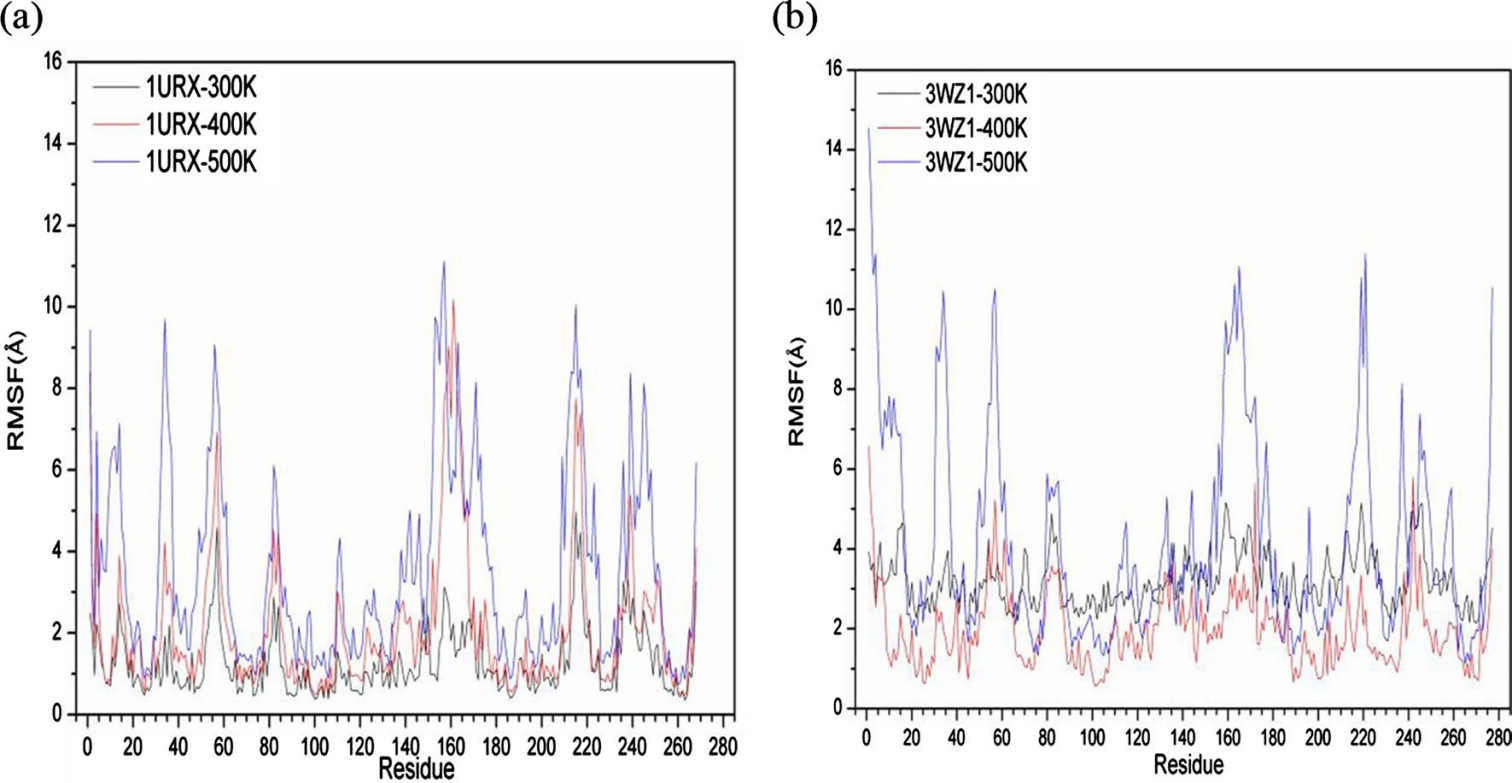
Fluctuations of RMSF for 1URX (**A**) and 3WZ1 (**B**) under different temperatures

Figure 3 shows the variations of the RMSF values of residues of the two homologous β-agarases (1URX and 3WZ1). The fluctuation level increases significantly from 300 to 400 K and further hugely increases at 500 K for 1URX. The average RMSF values are 1.27 Å, 2.10 Å, and 3.55 Å for 1URX at 300 K, 400 K, and 500 K. Although the fluctuation level increases significantly from 400 to 500 K for 3WZ1, the fluctuation level drops from 300 to 400 K. The average RMSF values are 3.19 Å, 1.96 Å, and 4.22 Å for 3WZ1 at 300 K, 400 K, and 500 K.

To further explore the reasons for the above observation, we also calculated the average value of SASA, VDW energies, and the average number of protein interior and protein–water intermolecular hydrogen bonds of 1URX and 3WZ1 at 300 K, 400 K, and 500 K. As we expect, the SASA increases from 300 to 400 K and 500 K, which is consistent to the increase of RMSD and RMSF with temperature. Other evaluation indexes including protein–protein H-bond, protein-solvent H-bond, VDW-force (protein), and VDW-force (protein-water) then generally decrease with simulation temperature, as shown in Table 1. We also found that 3WZ1 has more protein–protein H-bond and protein-solvent H-bond than 1URX.

### Evolution of secondary structure

**Fig. 4:**
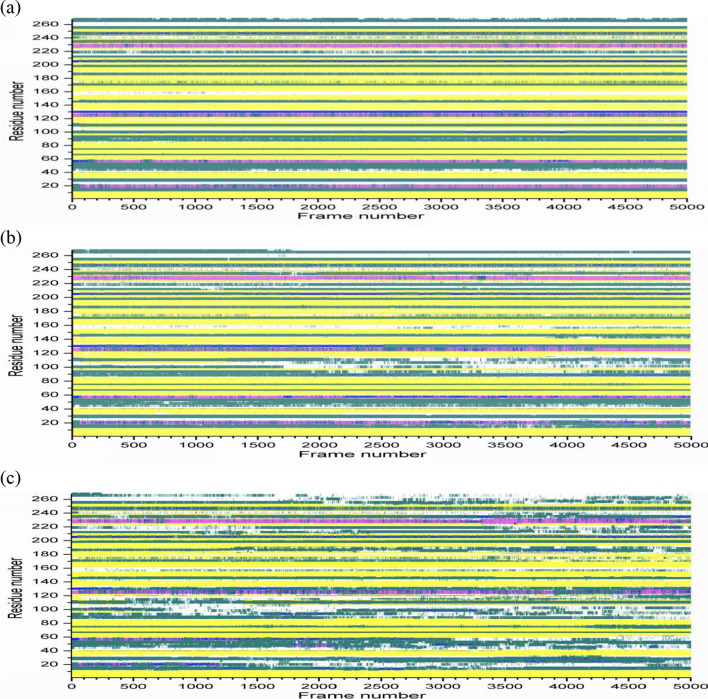
Secondary structure variations of 1URX at different temperatures. A: 300 K; B: 400 K; C: 500 K. The x-axis shows frame number (trajectory number), the y-axis shows amino acid sequence number. Cyan, yellow, tan, purple, blue, red and white represent the secondary structure of T (Turn), E (β- sheet), B (Isolated bridge), H (Alpha helix), G (3–10 Helix), I (Pi-helix) and C (coil) respectively. Values have been calculated with the use of backbones

**Fig. 5:**
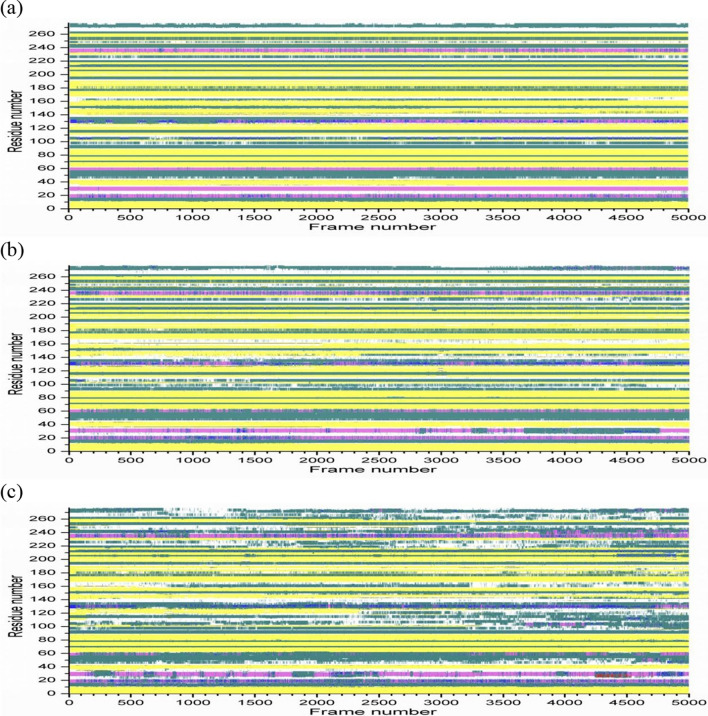
Secondary structure variations of 3WZ1 at different temperatures. A: 300 K; B: 400 K; C: 500 K. The x-axis shows frame number (trajectory number), the y-axis shows amino acid sequence number. Cyan, yellow, tan, purple, blue, red and white represent the secondary structure of T (Turn), E (β-sheet), B (Isolated bridge), H (Alpha helix), G (3–10 Helix), I (Pi-helix) and C (coil) respectively. Values have been calculated with the use of backbones

The secondary structures evolution of 1URX and 3WZ1 along with time can give us a further understanding of their structural fluctuation. The structural transitions of the two β-agarases at different temperatures were showed in Figs. 4 and 5. Briefly, the two β-agarases maintain folded at 300 K and 400 K, indicating that their crystal structures are similar to the original state. At the temperature of 300 K (Figs. 4A and 5A), the secondary structures of 1URX and 3WZ1 have rarely changed in the simulation process and have minor changes at 400 K (Figs. 4B and 5B). With higher temperature at 500 K, the structures of the two β-agarases have unfolded (Figs. 4C and 5C). The changes of the secondary structures were larger, and their behavior varied significantly. Thus, the structures started to lose their original secondary structure elements along with time at high temperature. Our results present significant structural variations in 4 helices, 12 sheets and 11 coils for 1URX and 4 α-helices, 6 β sheets and 10 coils for 3WZ1 at 500 K.

### Fluctuation level of residues

**Fig. 6:**
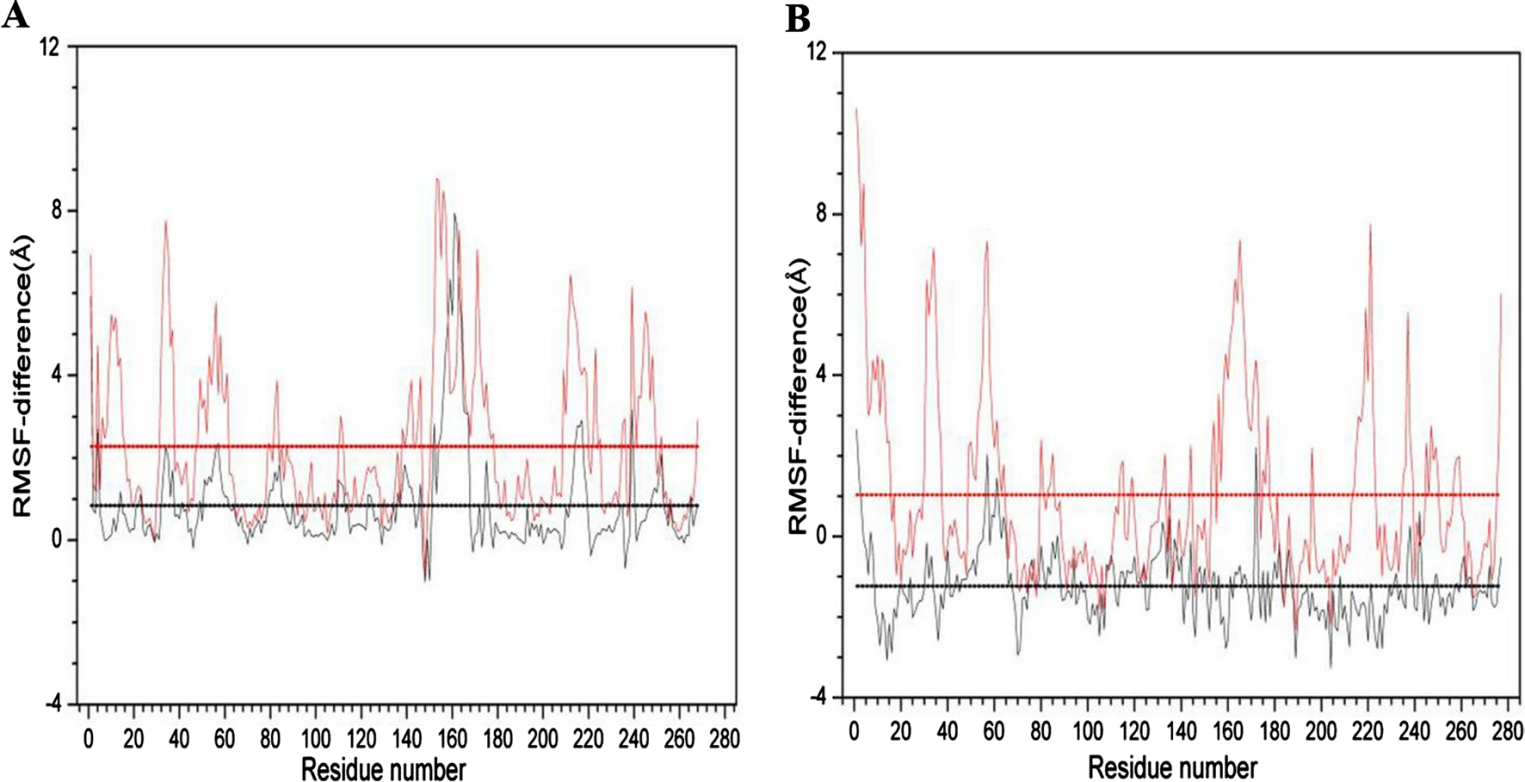
The RMSF-DIFF as a function of residue number in 1URX (**A**) and 3WZ1 (**B**). The black broken line indicates that the RMSF values of 400 K subtracted from the corresponding profile of 300 K, and the thick black lines are their averages. The broken red line indicates that the RMSF values of 500 K subtracted from the corresponding profile of 300 K, and the thick red lines are their averages

The residues with high fluctuation levels and their location can be identified through their RMSF-difference values (Fig. 6). We found the thermal sensitive regions of 1URX were residues at positions of 1, 4, 14, 32–37, 49, 51–61, 79–80, 82–83, 111–112, 138–142, 146, 152–167, 172, 175–176, 212–219, 235, 239–240, 248–249, 252, and 268. The thermal sensitive residues of 3WZ1 are residues 1–8, 31–33, 49–64, 80–81, 83–85, 114–115, 119, 131–132, 144, 153–154, 156, 161, 163–168, 172–173, 175–177, 181, 221, 237–239, 245, 249–250, 258, and 276–277. The thermostable regions of 1URX are only residues at positions of 29 and 148. The thermostable regions of 3WZ1 are residues at the positions of 18–23, 25, 38–40, 45–48, 66, 69–79, 89–110, 116–117, 121–126, 134, 136–138, 140–142, 146, 149, 151–152, 174, 180, 182–185, 187–195, 197–207, 209–212, 224, 226, 228–231, 233–234, 240, 251–253, 262–271, and 273–274. The above results indicate that there are more thermostable regions in 3WZ1 compared with 1URX.

Combining with the previous alignment results and the differences of secondary structure, we confirmed the residues (Phe32, Asn33) with large RMSF values in β sheet (residues 31–33) displayed thermal instability in 1URX, while the residue (Ser245) with large RMSF value and residue Asp240 with negative RMSF-DIFF in α helix (residues 240–245) of 3WZ1 displayed thermal sensitive and thermostable respectively. According to the previous results of the structural comparison (“Sequence alignment and structural comparison” Section), the residue Ser36 is one of the four differences of 1URX, and the residues (Ala2, Gly83, Ser245 and Ile277) are those of the eight differences of 3WZ1 displayed large RMSF values, indicating these residues are thermostable. Other residues, Pro141 and Gly142, are two of the eight differences contributing to the thermostability of 3WZ1. These results could illustrate that these specific residues should play important roles in thermostability.

### Distance and number of salt bridge

**Fig. 7:**
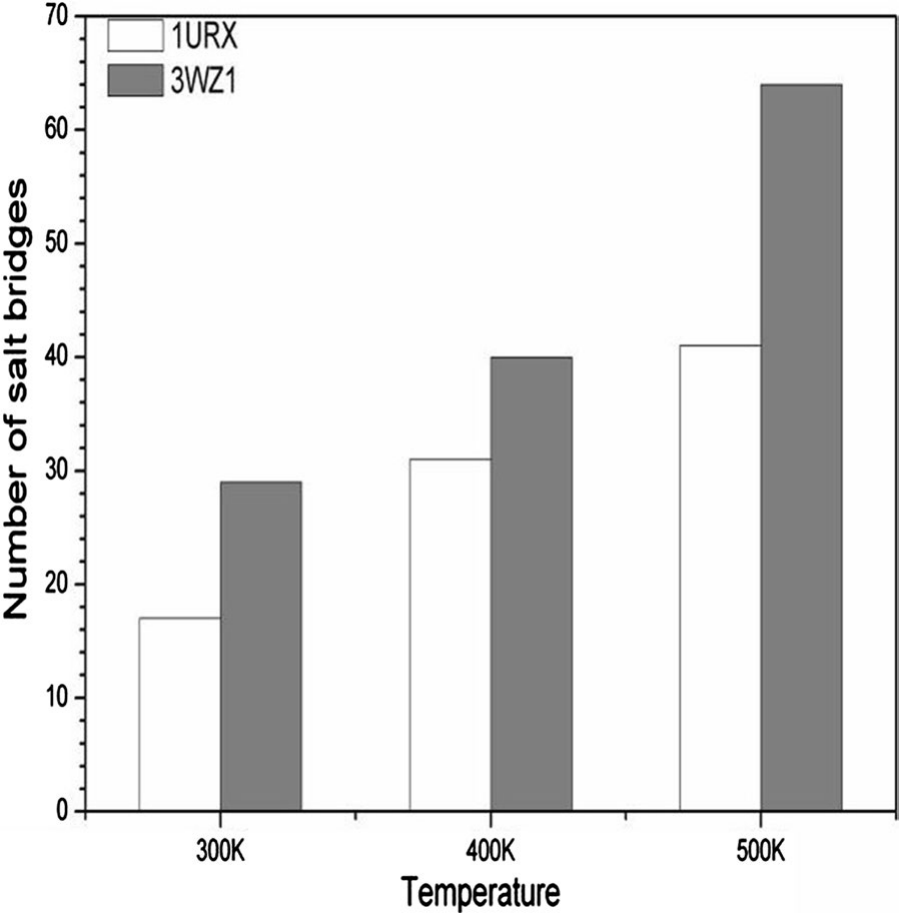
Number of salt bridges in 1URX and 3WZ1 at different temperatures

**Fig. 8:**
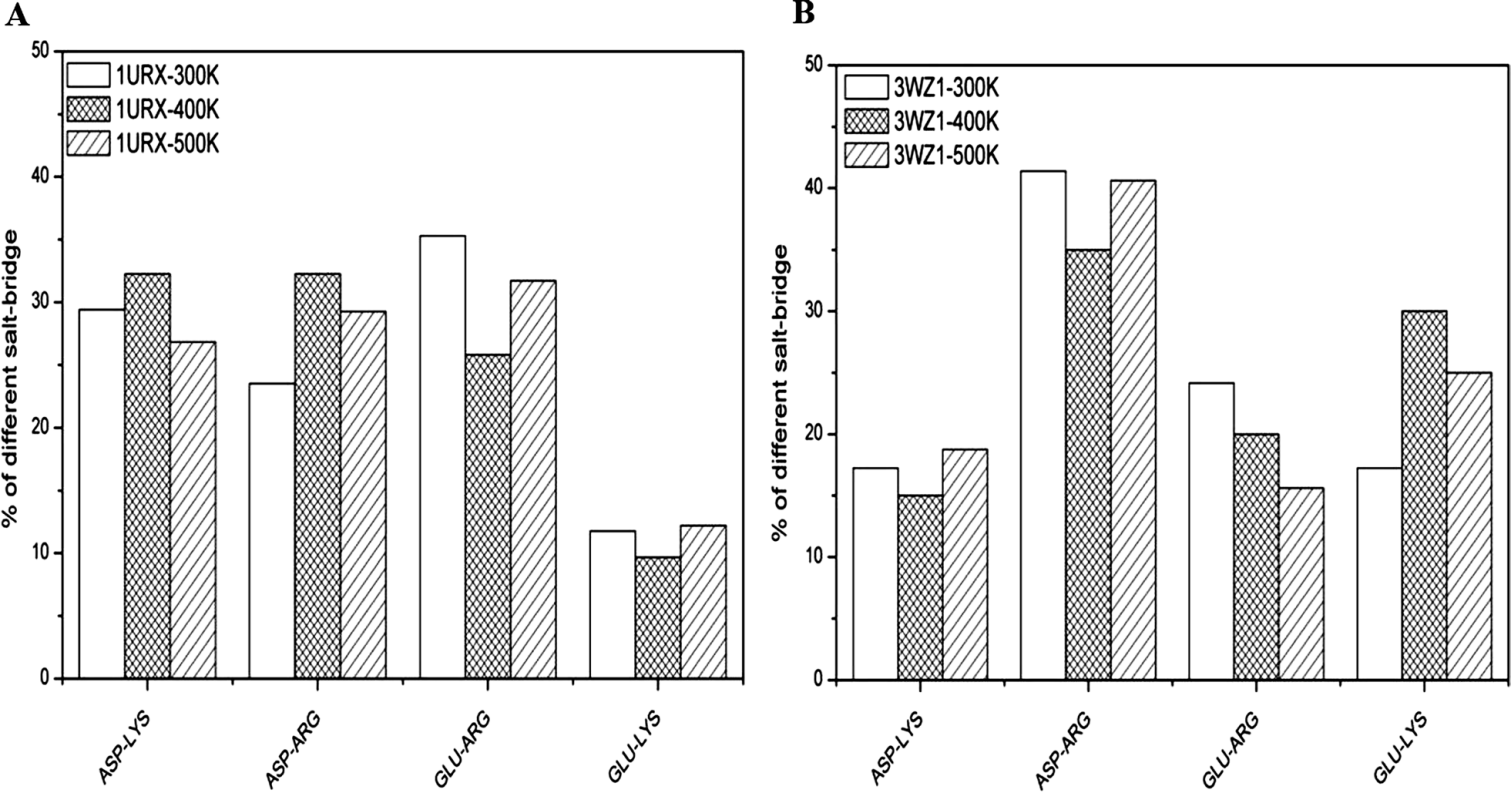
The variation of salt bridges in 1URX (**A**) and 3WZ1 (**B**) at different temperatures

To further explore the stabilization mechanism of salt bridges on the thermostability of 1URX and 3WZ1, all possible N(Lys/Arg)-O(Asp/Glu) interatomic distances and the number of these salt bridges were computed, as shown in Fig. 7. The salt bridges increased significantly with temperatures from 300 to 500 K, indicating the new salt bridges formed exceed the disrupted ones during the denaturation of 1URX and 3WZ1 in high temperatures of 400 K and 500 K. The different variations of salt bridges at the three temperatures are shown in Fig. 8.

The number of different kinds of salt bridges fluctuates with the increasing temperature, while salt bridges between Glu and Arg in 3WZ1 consistently decrease with the temperature. We thus infer that the salt bridge between Glu and Arg in 3WZ1 is thermally unstable. Further investigation showed that salt bridge interaction gradually weakened as the temperature increased. For example, the average distance of the salt bridge between Asp168 and Arg208 was 3.4 Å at 300 K in 3WZ1, while it hugely increased to 8.4 Å at 500 K, indicating that the salt bridge is thermally unstable.

In addition, the newly formed salt bridges of some residues were subjected to static affinities from other carboxyl groups; thereby, these interactional residues are unstable and easily alterable. For example, the average distance of the salt bridge between Glu62 and Arg63 was 5.3 Å at 300 K in 3WZ1. It changed to 5.9 Å at 500 K. Moreover, Glu62 simultaneously encountered static affinities from residue Arg59 (average distance of 9.2 Å) and residue Lys113 (average distance of 7.5 Å). In addition, the interaction of several salt bridges became strengthened or formed new salt bridges while the temperature increased. For example, the average distance of the salt bridge between Glu128-Arg158 was 9.8 Å at 300 K in 3WZ1, and it changed to 3.9 Å at 500 K, illustrating that the enhanced salt bridge can resist elevated temperature and improve the thermostability of β-agarases.

We further analyzed and compared the salt bridge under the different simulation temperatures and found that several salt bridges remain in the same state all the time. They were Asp26-Lys44, Asp200-Arg184, Asp250-Lys253, Asp122-Lys95 and Glu34-Arg37 in 1URX, while Asp168-Arg208, Glu60-Lys80, Glu127-Lys229, Asp252-Arg79, Asp257-Arg260 and Glu128-Arg158 in 3WZ1. Their average distances can be found in Table 2. Overall, these salt bridges listed in Table 2 should play critical roles in the thermostability of the two β-agarases.

## Discussion

In this study, we carried out MD simulations on two β-agarase monomeric crystal structures (1URX and 3WZ1) at 300 K, 400 K, and 500 K to understand the mechanism of thermostability of β-agarase by analyzing its structure. Our sequence alignment and structural comparison results demonstrate that 1URX and 3WZ1 shared homology to the members of the GH16 family. Further analysis shows that there are four different sites (Asn23, Asn36, Gly140 and Thr237) in 1URX and eight different sites or regions (Ala1, Pro33, Gly82, Pro140 ~ Gly141, Lys177, Gln213, Ser244 ~ Asp247 and Ile276 ~ Asn277) in 3WZ1. The residues within the above sites or regions play an important role in thermostability of 1URX and 3WZ1.

As one of mesophile enzymes, 1URX has a higher thermal sensitivity than 3WZ1, which is one of thermophile enzymes. This can be verified by the comparison of the variation of RMSD values between 1URX and 3WZ1 when the temperature increases from 300 to 400 K. Also, we found more thermostable regions exist in 3WZ1 compared with 1URX. For example, there are only two thermostable residues at positions of 29 and 148 in 1URX, but more than 50 residues are identified to be thermostable in 3WZ1.

The generally consistent variations of the index values in Table 1 indicate that the structures of 1URX and 3WZ1 tend to be unstable with increasing temperatures up to 500 K. Specifically, the structures of the two β-agarases become unfolded at high temperature of 500 K, while maintain folded at 300 K and 400 K. As we can see from Fig. 2A and B, when the temperature is 400 K, the RMSD value of 1URX is higher than 3WZ1, indicating that the unfolding of 3WZ1 occurred much later than the mesophile 1URX. Note the unfolding phenomenon of β-agarase is consistent with the earlier experimental studies regarding the β-agarase thermostability (Mahanta et al., 2015).

We found that most of hydrophilic residues in 3WZ1 contains more intramolecular hydrogen bonds, which can induce hydrophobic interactions, maintain thermostability and also increase the fractional polar surfaces of protein (Vogt et al., 1997; Ramos et al., 2018). Previous studies also show that hydrogen bonds and hydrophobic interactions between protein residues are responsible for their stability differences (Ramos et al., 2018; Fujii et al., 2021). Takagi et al. (2015) speculated that proline residues might also increase protein thermostability as they were uniformly distributed on the protein surface of *Mt*AgaA. But, no verification or comparison with other studies was provided. Generally, the above observations indicate that the residues of 3WZ1 with more excellent stability are the major contributors to their thermostability.

In addition, previous studies and salt bridge interactions results indicated that certain salt bridges are not conducive to the thermostability of β-agarases (Vogt et al., 1997). This result means the thermostability of 1URX and 3WZ1 could be increased by optimizing residues at these salt bridge sites. The residues and regions with high RMSFs could be sensitive to high temperature; this thermal sensitivity can be one of the major contributors to initial thermal denaturation. Improving the stabilities of these residues or regions at high temperature could improve the thermostability of β-agarases. Lee et al. (2011) reported that the catalytic activity and thermostability of the β-agarase AgaA gene from Zobellia galactanivorans could be improved after performing site-directed mutagenesis on the β-agarase AgaA gene. Alternatively, the thermal sensitive residues and regions can be deleted for achieving thermostability. For example, the α helix (residues 240–245) in β-agarase 3WZ1 could be removed to increase its tolerable temperature. We can also introduce suitable residues to form stable salt bridges in unstable regions or replace the poor thermostability amino acids with stable ones. The above proposed strategies may be initiated by using MD simulation to recognize the critical structural elements such as salt bridges and further implemented by modifying the residues at the salt bridge sites that are not conductive to the thermostability of β-agarases. Agarases with good thermostability can have great potential in industrial applications.

## Data Availability

Data and materials available on request from the authors.
